# Identity information content depends on the type of facial movement

**DOI:** 10.1038/srep34301

**Published:** 2016-09-29

**Authors:** Katharina Dobs, Isabelle Bülthoff, Johannes Schultz

**Affiliations:** 1 Department of Human Perception, Cognition and Action, Max Planck Institute for Biological Cybernetics, Tübingen, Germany; 2 Université de Toulouse, Centre de Recherche Cerveau et Cognition, Université Paul Sabatier, Toulouse, France; 3 CNRS, Faculté de Médecine de Purpan, UMR 5549, Toulouse, France; 4 Division of Medical Psychology and Department of Psychiatry, University of Bonn, Bonn, Germany

## Abstract

Facial movements convey information about many social cues, including identity. However, how much information about a person’s identity is conveyed by different kinds of facial movements is unknown. We addressed this question using a recent motion capture and animation system, with which we animated one avatar head with facial movements of three types: (1) emotional, (2) emotional in social interaction and (3) conversational, all recorded from several actors. In a delayed match-to-sample task, observers were best at matching actor identity across conversational movements, worse with emotional movements in social interactions, and at chance level with emotional facial expressions. Model observers performing this task showed similar performance profiles, indicating that performance variation was due to differences in information content, rather than processing. Our results suggest that conversational facial movements transmit more dynamic identity information than emotional facial expressions, thus suggesting different functional roles and processing mechanisms for different types of facial motion.

In real life our faces are constantly moving, expressing our state of mind and enabling us to socially interact with others. Humans quickly extract and interpret even subtle social information conveyed by facial movements. While classic models of face perception assume that facial motion processing is largely independent of identity processing^1^,^2^, characteristic facial movements can help to recognize faces^3^,^4^,^5^,^6^. To date, the exact role of facial motion as a cue for identity is still under debate^6^,^7^. In particular, essential unanswered questions include how much information about identity is conveyed by different types of facial movement, and how efficiently the face perception system extracts these cues. Here, we study identity in facial motion from the viewpoint of information transmission: we consider the face as a sender of dynamic information, of which identity information is one aspect, and the observer as a decoder of this information^8^.

From the sender’s side, different types of facial movements may differ in their functional role. According to the sensory regulation hypothesis, emotional facial expressions not only convey a mental state, but also alter the sensory systems to prepare for perception and action^9^,^10^. Evidence suggests that, for example, eye widening in a fearful face makes it easier for the person experiencing fear to detect threats in the periphery of the visual field^9^,^11^. Despite this nonsocial sensory function, emotional facial expressions have co-evolved with social-emotional communication. For example, the same wide eyes characteristic of an expression of fear makes it also easier for an observer to discriminate the eye gaze direction of the person experiencing fear, helping to locate the fear-inducing stimulus^11^. To fulfill these sensory and social regulatory functions, expressions of basic emotions must therefore be expressed unequivocally^12^,^13^,^14^, and thus are likely to contain little identity-specific content^15^. In contrast, the facial movements used in a conversational setting originate in social communication^10^ and serve communicative functions beyond signaling emotions, such as signaling speech content through lip movements^16^. Indeed, conversational facial movements are more subtle and personal, and may thus allow individual nuances about the mental state of the speaker to be conveyed^17^,^18^,^19^. In line with these findings, previous studies reporting identity information in facial motion mainly used conversational and speech-related facial movements^3^,^20^. However, to our knowledge, no study systematically investigated the amount of identity information contained in different types of facial movement.

From the observer’s side, face perception involves simultaneous processing of the identity and expression of a face. For emotional facial expressions, quickly interpreting the signal being transferred is more important than recognizing who is sending it. Such an optimized processing of emotional signals, as observed for static faces^13^, may hinder the extraction of identity information. However, for more complex facial expressions, displayed by moving real-life faces, processing of identity and expression may influence each other to a variable degree, and may depend on the type and emotional content of the facial movements^4^,^7^,^21^,^22^,^23^.

This study aimed to investigate whether the amount of identity information coming from the sender and the use of this information by the observer depend on the type of facial movement. We recorded facial movements from four different actors in three different natural settings, evoked through a method-acting protocol: (1) purely emotional facial expressions without interaction, (2) emotional facial expressions occurring in a social interaction, and (3) conversational facial movements with little affective content, occurring in a social interaction. While these three types of facial movements do not represent clearly separated categories, instructions were sufficiently different to yield clearly separable types of facial expressions. Using a recent motion capture and animation system^1^,^2^,^24^, we animated a single avatar head with the recorded movements and asked observers to match the four identities for each type of facial movement in a two-alternative forced-choice, delayed match-to-sample task. To quantify the transmitted identity information in the facial movements, we built model observers based on the motion characteristics and compared their performance to human observers.

## Results

### Model observer results

**Figure 1:**
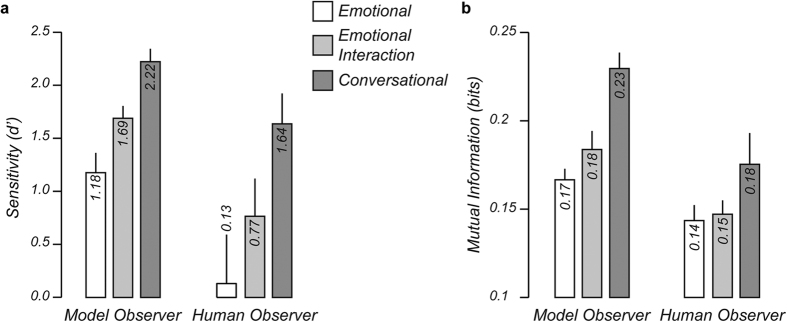
(**a**) Mean sensitivity (d’) to the identity of the actor performing the facial movements, as a function of facial movement type, across human observers (n = 14) and model observers corresponding to each human observer. A sensitivity of 0 indicates chance performance. (**b**) Mutual information (bits) as a function of facial movement type across human observers (n = 14) and model observers. Error bars indicate 95% confidence interval (CI).

Model observer data revealed above-chance performance in matching the identity of the actor in all three types of facial movement (Fig. 1A, left side; emotional: t(13) = 13.60, p < 0.001, emotional interaction: t(13) = 31.45, p < 0.001, conversational: t(13) = 39.31, p < 0.001). The sensitivity was lowest for purely emotional facial movements, higher in emotional facial movements in social interactions and largest for conversational facial movements (emotional vs. emotional interaction: t(13) = −5.14, p < 0.001; emotional vs. conversational: t(13) = −10.48, p < 0.001; emotional interaction vs. conversational: t(13) = −5.34, p < 0.001; p values Bonferroni-corrected). Confirming these results, mutual information (i.e., measured in bits) increased in the same order (Fig. 1B, left side). Mutual information was lower for emotional facial movements than for emotional facial movements in social interactions and largest for conversational facial movements (emotional vs. emotional interaction: t(13) = −2.98, p = 0.006; emotional vs. conversational: t(13) = −10.97, p < 0.001; emotional interaction vs. conversational: t(13) = −7.99, p < 0.001; p values Bonferroni-corrected). We therefore conclude that emotional facial movements contain less identity information than conversational facial movements. How much of this information is used by the human face perception system?

### Human observer results

Human observers were able to discriminate identities based on conversational facial movements and emotional facial movements occurring in social interactions (Fig. 1B, right side; emotional interaction: t(13) > 4.66, p < 0.001; conversational: t(13) = 12.35, p < 0.001), but not based on purely emotional facial movements (t(13) = 0.61, p = 0.551). Just as for model observers, sensitivity was lowest for emotional and highest for conversational movements (emotional vs. emotional interaction: t(13) = −2.56, p = 0.017; emotional vs. conversational: t(13) = −6.07, p < 0.001; emotional interaction vs. conversational: t(13) = −3.51, p = 0.001; p values Bonferroni-corrected). Mutual information increased in the same order (Fig. 1B, right side), with the largest values for conversational and the smallest for emotional facial movements (emotional vs. conversational: t(13) = −3.92, p < 0.001; emotional interaction vs. conversational: t(13) = −3.48, p = 0.002; p values Bonferroni-corrected). However, mutual information did not significantly differ between purely emotional and emotional facial movements in social interactions (t(13) = −0.44, p = 0.664). These results suggest that more identity information can be extracted from conversational than emotional facial movements, which is in line with the higher amount of identity information contained in conversational facial movements compared with emotional ones. However, do humans use identity information equally efficiently across types of facial movement?

### Relationships between human and observer performance

As model observers were programmed to use the same processing strategy in all trials, we directly compared human and model observer performance across movement types. Performance varied with both factors (main effects: movement: F(2,52) = 67.17, p < 0.001; observer: F(1,26) = 45.73, p < 0.001), confirming the performance differences across movement types described earlier and revealing that human observers are less sensitive to identity information than model observers. Crucially however, the difference in performance between human and model observers did not change across movement types (no significant interaction between movement type and observer: F(2,52) = 1.59, p = 0.213). These results suggest that humans use identity information equally efficiently across movement types. Analysis of mutual information confirmed these results: the percentage of mutual information used by human observers did not differ across facial movement types (mean and SEM: emotional: 86.33 ± 1.51%, emotional in social interaction: 81.00 ± 1.37%, conversational: 76.40 ± 3.10%; main effect: F(2,26) = 2.88, p = 0.0739). We thus conclude that differences in human observers’ sensitivity to identity information carried by different types of facial movements are mainly due to the amount of identity information transmitted by these facial movements, and not due to processing strategies.

### Variability in facial movements

We asked whether any differences in sensitivity between types of facial movements could be explained by differences in the variability between and within actors for different types of facial movement. For example, sensitivity to identity would be high when one actor’s facial movements are similar regardless of his expression, yet maximally different from the other actors’ facial movements. For each type of facial movement, we calculated the variability ratio of all possible facial movement combinations between the same actor (“within” variability) and different actors (“between” variability). Variability ratios differed between types of facial movement (mean ± SEM emotional: 1.19 ± 0.09, emotional interaction: 1.20 ± 0.10, conversational 2.65 ± 0.75; F(69) = 6.25, p = 0.003). The ratio was largest for conversational facial movements (emotional vs. conversational: t(23) = −3.06, p = 0.004; emotional interaction vs. conversational: t(23) = −3.04, p = 0.004; p values Bonferroni-corrected), but did not differ between emotional and emotional in social interaction (t(23) = −0.02, p = 0.982). This result is consistent with the results of the behavioral and model observer analyses and suggests that conversational facial movements contain most identity information due to high variability between actors and low variability within actors (i.e., actors move most differently from each other when they perform conversational expressions).

### Controlling for low-level differences in overall amount of facial motion

**Figure 2:**
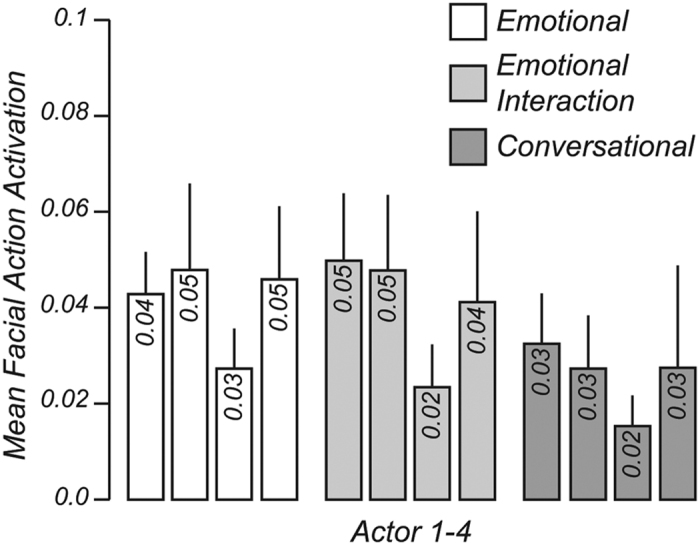
Controlling for differences in the amount of facial motion across actors. Mean facial action activation is reported as a function of facial movement type and actor performing the movements. There was no significant difference in action activation across facial movement types or actors. Error bars indicate SEM.

To exclude the possibility that the differences in identity information across types of facial movements revealed in the previous analyses are due to low-level differences in the overall amount of facial motion, we searched for differences in mean activation of facial actions across facial movement types and actors (Fig. 2). The mean activation of facial actions neither differed between facial movement types (F(2) = 1.57, p = 0.209) nor between actors (F(3) = 1.33, p = 0.264). Moreover, crucially, the difference in mean facial action activation between actors did not change across facial movement types (F(6) = 0.07, p = 0.999). This analysis thus suggests that the amount of identity information cannot be simply explained by differences in the overall amount of facial motion.

## Discussion

Our findings suggest that the amount of identity information conveyed by facial motion varies with the type of facial movement, that human observers do not decode all of this information, but that they decode similar proportions of this information across movement types. We based our study on the theoretical viewpoint that emotional and conversational facial movements may differ in their functional role: while emotional facial expressions may be primarily optimized for perception and action^9^,^10^,^11^, facial movements occurring in everyday conversations facilitate social communication^10^,^17^,^18^,^19^,^25^, and may thus convey more identity information. In line with this hypothesis, using evidence from both human and model observers, we found that the human face transmits more dynamic identity information in conversational than in emotional facial movements.

Our results corroborate and extend existing evidence that facial motion can be used as a cue to identity^3^,^4^,^5^,^6^,^20^,^26^. Previous studies reported identity information for particular types of facial motion such as basic facial movements^5^,^13^,^27^, expressive and talking movements^3^,^4^,^7^,^20^,^22^,^23^ or purely speech movements^28^. While all these studies evidenced the presence of identity information in facial movements, differences in stimuli and task across these studies did not allow for a comparison between amounts of identity information. Using the same task and stimulus generation method, we showed that basic emotional facial movements contained least, whereas conversational facial movements contained most, identity information. However, the absolute amount of identity information we obtained from our motion-captured data is almost certainly underestimated compared to the information contained in real life faces. This is due to possible information loss during the retargeting and animation procedure, and to the fact that we removed rigid head motion which has been previously reported to carry identity information^3^,^20^. Moreover, the use of a single avatar head may somewhat limit the generalizability of our findings to other facial forms. In future studies it would be interesting to manipulate the amount of identity information contained in facial form and facial motion in a controlled way to address the interaction between both types of information for identity perception. Taken together, our results show that the amount of identity information varies with the type of facial movement and, importantly, suggest different functional mechanisms underlying different types of facial movements.

The use of a state-of-the-art motion retargeting technique allowed us to compare human performance with that of model observers. The use of a model observer is a technical innovation that may improve our understanding of face processing by providing a quantification of the amount and content of the information available in facial movements. However, such model observer analyses can be implemented in different ways, which may influence the outcome of these analyses. For example, our model observer did not take into account any temporal correlations between activations of facial actions, which might have improved the performance of the model observer. While it may be useful to systematically compare different types of model observers and to relate those to human performance, this was out of the scope of our present study.

The ability to decode identity from emotional facial movements in social interaction and from conversational facial movements suggests an important role of speech movements for identity processing. Our results are consistent with previous studies reporting that facial speech can be used to recognize faces^3^,^28^ and might even be useful for biometric applications in person identification^15^,^29^. Moreover, studies investigating the bimodal link between voice and face perception suggest that the rhythm of speech may contain identity information^30^,^31^. Future studies could thus address which facial speech cues are idiosyncratic and if they might even be available cross-modally.

Identity information, however, is likely not only present in speech movements. While speech movements were contained in two types of facial movements: emotional in social interaction and conversational, both types clearly differed in their identity information content. At least two alternative mechanisms could explain this finding. The enhanced identity information content in conversational facial movements may be due to subtle idiosyncratic facial movements conveyed by conversational facial movements (e.g., such as an identity-specific raising of the eyebrows). Alternatively, the presence of emotional content in the emotional facial movements in social interactions may lead to processing strategies which are suboptimal to extract identity information^6^,^13^. However, the comparison between human and model observers’ performance did not reveal an interaction of facial movement type and observer type, suggesting that humans were equally efficient in extracting identity information across different types of facial movement. Thus, our data do not support differences in processing for the different types of facial movements, but rather support the presence of additional identity-specific facial movements in conversational facial expressions. However, future research is required to investigate which spatio-temporal characteristics of facial movements contain identity information.

While our results support the conclusion that emotional facial movements contain less identity information than conversational facial movements, the specificity of the stimuli and task used may limit the generalizability of our findings. Particularly, it would be interesting to repeat the experiment with more actors, different avatars, other tasks than a two-alternative forced-choice delayed match-to-sample, more exemplars for each type of facial movement, or to include more types of facial movements. Perhaps even more interesting could be to assess the use of identity in facial motion in more ecologically valid situations, such as natural videos, or full-body virtual reality setups. While technically challenging to a point much beyond the scope of the present study, such further studies would lead to a better understanding of the use of identity information in facial motion.

From an evolutionary perspective, our findings support interesting ideas about the emergence of different types of facial movement. Emotional facial expressions may have evolved under the pressure to optimize perception and action^9^,^10^,^11^ and to optimally convey emotional signals^13^. In contrast, observing conversational facial expressions may allow us to capture individual nuances in the way a person moves their face. This could allow us to estimate the person’s mental state better^18^,^19^,^32^, as it allows us to combine personal information we have about the person with more generally-applicable knowledge about mental states.

In summary, our findings help to understand the role of motion in identity processing, an essential function of the face processing system that is still poorly understood. While classic models of face perception propose that facial expression and identity are processed independently^1^,^2^, our findings suggest that this independence critically depends on the type of facial motion, thus providing substantial new constraints for such models. Our data, however, are consistent with recent models emphasizing a functional dissociation between form and motion rather than between identity and expression of faces^7^,^33^. Our findings thus raise interesting questions regarding the neural mechanisms underlying face perception and will hopefully stimulate further research investigating the effect of different types of facial motion on face perception.

## Material and Methods

### Participants

Fourteen participants (6 female; mean age: 30.2, SD: 7.5) were recruited from our local subject database and participated in the experiment. All observers were naive to the purpose of the experiment and had normal or corrected-to-normal vision. They provided informed written consent before the experiment and filled out a post-questionnaire thereafter. The study was approved by the ethics review board of the Max Planck Society and conducted in accordance with relevant guidelines and regulations.

### Stimuli and Display

**Figure 3:**
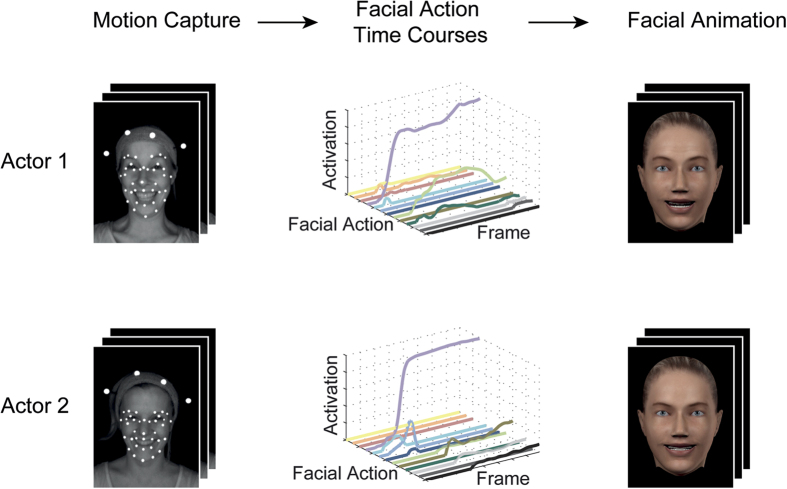
Schematic overview of the facial animation procedure, shown for the facial expression happiness. In the first step (motion capture, left), facial expressions are recorded from different actors using a marker-based motion capture system (41 markers shown as white dots). In the second step (facial action time courses, middle), the facial animation system decomposes the motion capture data of each facial expression into 30 time courses of facial action activation (e.g., eyebrow raising). Here a subset of 13 facial actions is depicted for demonstrative purposes. In the last step (facial animation, right), the time courses of facial action activation are used to animate a single 3D face model designed in Poser 8 (SmithMicro, http://poser.smithmicro.com/poser.html).

To create dynamic face stimuli that were identical in facial form but differed in facial motion, we used a motion-retargeting and facial animation procedure (Fig. 3). The animation procedure was as follows. First, we motion-recorded 12 different facial movements of four non-professional female actors using a marker-based optical motion capture system^3^,^4^,^5^,^6^,^24^. Facial movements were performed according to a method-acting protocol in which the actor was verbally given a specific background scenario designed to elicit the desired facial movements. This protocol is a widely accepted and common approach to elicit natural facial expressions^6^,^7^,^17^,^34^,^35^. Actors began each facial movement with a neutral expression. The 12 facial movements were recorded in three types of natural settings: “emotional” (i.e., purely emotional facial expressions), “emotional in social interaction” (i.e., emotional and facial speech movements occurring in a social interaction) and “conversational” (i.e., conversational and facial speech movements with little affective content occurring in a social interaction). Details of each facial movement and its corresponding exemplary scenario eliciting the movement are listed in Table 1. Second, we chose one female avatar head in Poser 8 (SmithMicro, Inc., Watsonville, CA, USA) and modified it, to give it an average appearance (e.g., the lips were made thinner). The resulting head was animated by each of the motion-recorded facial movements. To this end, we used a system that decomposes the recorded motion data into time-courses of meaningful facial action activation (e.g., eyebrow raising)^24^,^36^. The activation for each of the 30 facial actions^8^,^24^,^36^ during a recorded facial expression can range from 0 (no activation) to 1 (maximum intensity), thus representing the amplitude of this facial action at a given time point (see middle column of Fig. 3). Those time-courses of facial action activation were used to animate the same avatar head and hence ensured that animations were independent of an actor’s individual facial form. Finally, the 48 animations were rendered as Quicktime movies of 3.5 s duration (300 × 400 pixels, Codec H.264, 60 Hz) in 3ds Max 2012 (Autodesk, Inc., San Rafael, CA, USA).

Stimuli were presented and responses recorded using PsychToolbox 3 for Matlab (http://www.psychtoolbox.org)^9^,^10^,^37^. Observers were seated approximately 60 cm from a Dell 2407WFP monitor (24 inch screen diagonal size, 1920 × 1200 pixel resolution; 60 Hz refresh rate). Animation stimuli were scaled to a size of 9° × 12°.

### Design and Procedure

Prior to the experiment, we briefly familiarized observers with the stimulus material and the four different actors by presenting them video stimuli identical to those used in the experiment but showing the facial movement “surprise”. This facial movement had been recorded from each of the four actors and animated the same avatar head as the test stimuli. Furthermore, the facial movement “surprise” was not used in the main experiment to ensure that observers were equally familiar with all the facial movements shown in the main experiment. During the training, the presentation order of actors was randomized. After watching each stimulus up to five times, observers had to answer two questions about the actor (e.g., “How happy is this person?”) intended to get observers acquainted with the concept that different actors can animate the same avatar head. The training lasted about 10 minutes.

In the experiment, observers performed a delayed match-to-sample task on the stimuli, in three experimental blocks separated for each type of facial movement (i.e., emotional, emotional interaction, conversational). The order of blocks was randomized across observers. In each block (e.g., emotional block), observers first watched a video clip of one of the four facial movements recorded for this type of facial movement (e.g., happiness) as sample, followed by two video clips displaying a different facial movement of the same type (e.g., disgust) as test stimuli. One of these test stimuli was performed by the same actor as the sample, while the other one was performed by a different actor (left/right location was randomized). Observers had to choose which of the test stimuli was performed by the same actor as the sample. Each of the 12 possible actor combinations (e.g., actor 1 as sample paired with actor 2 as test stimuli) was randomly shown four times in each block for a total of 48 trials. Facial movements were randomly selected such that each of the 12 facial movement combinations (e.g., happiness as sample and fear as test stimuli) was shown once with each sample actor (e.g., once in the 12 trials in which actor 1 served as sample), and thus shown four times per block.

**Figure 4:**
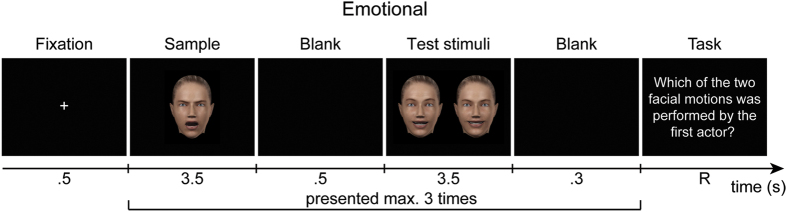
An example of the trial procedure of the experiment shown for emotional facial movements. The facial avatar was designed in Poser 8 (SmithMicro, http://poser.smithmicro.com/poser.html).

Figure 4 depicts an example of the trial procedure shown in an “emotional” block. Trials began with a central white fixation cross on a black background, followed by the sample stimulus. A black screen then appeared, followed by two test stimuli presented side-by-side, 6.7° to the left and right of fixation. This sequence was shown up to three times. A response screen appeared as soon as observers pressed a key (standard computer keyboard) or after the third presentation. The response screen remained until observers responded by pressing the left or right arrow key to choose the stimulus matching the sample. No feedback was provided. Up to three self-timed breaks every 12 trials were allowed per block and two 15-minute breaks between blocks (block time: 35–45 min, total experiment time: 120–160 min).

### Model Observer Simulation

To measure the identity information available in the spatio-temporal characteristics of facial movements, and therefore to be able to assess human use of this information, we built model observers corresponding to the human observers. That is, a model observer performed the same experiment as each of the human observers (the exact same trial sequence was used for the model and human, and this trial sequence differed across human observers). Model observer choices were computed as follows. First, we measured the similarity of facial action activation between each test stimulus a and b and the sample stimulus x (e.g., between happiness of actor 1 and anger of actor 1 and 2), respectively. To this end, we computed the distance between any facial movements a and x as the Euclidean distance between the mean activation of the 30 facial actions (e.g., eyebrows raising) used to create each stimulus:


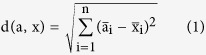


where n = 30 is the number of facial actions used and a_i_ represents the mean activation of facial action i in facial movement a across time (i.e., 210 frames). Second, we used Luce’s choice rule to compute the probability for a model observer to choose facial movement a given the two test stimuli a and b and sample x^9^,^11^,^38^. We have previously used this rule successfully to predict the choice probability in a delayed match-to-sample task using facial animations^11^,^24^. In Luce’s choice rule, the probability of choosing facial movement a (response r_a_) given stimuli a and b and sample x can be expressed as:


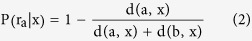


where d(a,x) is the similarity between facial movement a and the sample x, and d(b,x) is the corresponding similarity between facial movement b and the sample x (see equation 1). Third, for each trial, we simulated a model observer’s choice by comparing the choice probability P(r_a_|x) with 0.5 (i.e., P(r_a_|x) > 0.5 indicates the choice of match a, whereas P(r_a_|x) < = 0.5 indicates choice of match b). Note that while this model observer analysis is designed to obtain an objective measure of the amount of identity information, it may not necessarily be the upper bound of identity information contained in the stimuli; for example, taking another distance measure into account, e.g. one based on the dynamics of facial action activation^39^, or another type of choice rule, might improve performance.

### Data Analysis

#### Human and model observer data analysis

We assessed human sensitivity to identity information by calculating the d’ value for the three types of facial movement. We used the differencing rule for match-to-sample tasks to calculate d’; this assumes that observers compare each of the test stimuli a and b with the sample x and base their decision on these two comparisons^12^,^13^,^14^,^40^. We applied the same formula to the model observer data. T-tests were used to assess whether sensitivity was significantly different from chance and to reveal differences in sensitivity between types of facial movement. A two-way mixed measures ANOVA with facial movement type as within factor and observer type as between subjects factor assessed behavioral effects of facial movement type and differences between human and model observers.

#### Mutual information analysis

To quantify the amount of information about an actor’s identity contained in the stimuli, we measured the mutual information for each type of facial movement in both human and model observers according to Shannon information theory^15^,^41^. Here, mutual information (*MI*) is defined as the reduction of uncertainty about an actor’s identity *i* obtained by knowing the observer’s response *r* (also called equivocation^42^):


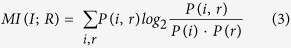


where *P(i, r)* is the joint probability of observing the response *R* = *r* together with the stimulus identity *I* = *i*. This joint probability can be estimated from the empirical confusion matrices (e.g., indicating how often actor 1 was identified as actor 1, actor 2, etc.) obtained for each observer and each facial movement type. Mutual information is measured in bits; if stimulus identity and responses are independent, mutual information is 0, otherwise it is positive. T-tests were used to reveal differences in mutual information between types of facial movement. To test for differences in how information is exploited between human and model observers across the three types of facial movements, we calculated the percentage of the available information used by human observers by dividing the mutual information measured in human observers by the corresponding values measured in model observers. A one-way ANOVA was used to test whether these percentages differ across facial movement types.

#### Analysis of variability in facial movements

To assess whether variability in facial movements could account for differences in performance between the three types of facial movements, we analyzed the similarity of facial movements between and within actors. Specifically, we assessed whether some types of facial movements were performed more similarly by all actors, which would imply that they contained less identity information. For each type of facial movement, we used the similarity measure used for the model observer (see Equation 1) to calculate the similarity between two facial movements of different actors (e.g., happiness of actor 1 and anger of actor 2) and of the same actor (e.g., happiness and anger of actor 1). For each actor and type of facial motion (emotional, emotional interaction, conversational), we obtained one similarity value for each of the six possible facial movement combinations (e.g., for emotional: happiness and anger) as within-actor variability. To obtain a corresponding value of the between-actor variability for each facial movement combination (e.g., happiness and anger), we averaged the similarities across the six possible actor combinations (e.g., actor 1 and actor 2). We then calculated the ratio of between- versus within-actor variability: the mean of between variability divided by the mean of within variability, for each actor and facial movement combination. The larger this ratio, i.e. the larger the difference in facial movements between actors compared to within actors, the more identity information should be contained in a type of facial movement. A one-way ANOVA and two-tailed t-tests were used to test for differences between the 24 ratio values (4 actors × 6 facial movement combinations) for each facial movement type.

#### Analysis of overall amount of motion

To exclude the possibility that differences in identity information across types of facial movements are due to differences in the overall amount of facial motion (e.g., small vs. large facial movements), we analyzed the mean activation of facial actions. Facial movement types and actors might differ in their overall amount of motion, and differences between actors in their overall amount of motion might depend on facial movement type. To assess whether there were any differences across actors or facial movement types in the overall amount of motion, we calculated the mean activation of each facial action over time and averaged these values across the four facial movements recorded for each type of facial movement. This resulted in 30 mean facial action activation values for each actor and each facial movement type. We used a two-way ANOVA to test for differences due to actor or facial movement type on the overall amount of facial motion.

## Additional Information

**How to cite this article**: Dobs, K. *et al*. Identity information content depends on the type of facial movement. *Sci. Rep.* **6**, 34301; doi: 10.1038/srep34301 (2016).

## Figures and Tables

**Table 1:** Overview of the 12 recorded facial movements, their type and exemplary scenarios eliciting the movement.

Type	Facial movement label	Scenario eliciting the movement
Emotional	Anger	You are very hungry and realize that someone took the food which you were looking forward to all day.
	Disgust	After you come home from a journey, you find mouldy food in your fridge.
	Fear	While cycling downhill, you suddenly realize that your brakes do not work.
	Happiness	You are laughing about a funny scene in a movie.
Emotional Interaction	Anger_Social	You are yelling at someone because he took your food without asking.
	Disgust_Social	You tell a friend how disgusting you think mouldy food is.
	Fear_Social	You are very frightened because you saw something in the forest at night and you try to warn a friend.
	Happiness_Social	You tell a friend about an exam that you just passed and that you are very happy about.
Conversational	Introduction_Casual	You introduce yourself to someone on a party.
	Introduction_Professional	You introduce yourself to your prospective employer.
	Farewell_Casual	You say goodbye to a friend on a party.
	Farewell_Professional	At the end of a job interview, you say goodbye to your prospective employer.

## References

[b1] Bruce V. & Young A. Understanding face recognition. Brit. J. Psychol. 77, 305–327 (1986). 3756376 10.1111/j.2044-8295.1986.tb02199.x

[b2] Haxby J. V., Hoffman E. A. & Gobbini M. I. The distributed human neural system for face perception. Trends Cogn. Sci. 4, 223–233 (2000). 10827445 10.1016/s1364-6613(00)01482-0

[b3] Girges C., Spencer J. & O’Brien J. Categorizing identity from facial motion. Q. J. Exp. Psychol. B. 68, 1832–1843 (2015). 10.1080/17470218.2014.993664 25687732

[b4] Lander K. & Chuang L. Why are moving faces easier to recognize? Vis. Cogn. 12, 429–442 (2005).

[b5] Knappmeyer B., Thornton I. M. & Bülthoff H. H. The use of facial motion and facial form during the processing of identity. Vision Res. 43, 1921–1936 (2003). 12831755 10.1016/s0042-6989(03)00236-0

[b6] O’Toole A. J., Roark D. A. & Abdi H. Recognizing moving faces: A psychological and neural synthesis. Trends Cogn. Sci. 6, 261–266 (2002). 12039608 10.1016/s1364-6613(02)01908-3

[b7] Lander K. & Butcher N. Independence of face identity and expression processing: exploring the role of motion. Front. Psychol. 6, 255 (2015). 25821441 10.3389/fpsyg.2015.00255 PMC4358059

[b8] Jack R. E. & Schyns P. G. The Human Face as a Dynamic Tool for Social Communication. Curr. Biol. 25, R621–R634 (2015). 26196493 10.1016/j.cub.2015.05.052

[b9] Susskind J. M. . Expressing fear enhances sensory acquisition. Nat. Neurosci. 11, 843–850 (2008). 18552843 10.1038/nn.2138

[b10] Darwin C. The Expression of the Emotions in Man and Animals (Fontana Press, 1999).

[b11] Lee D. H., Susskind J. M. & Anderson A. K. Social Transmission of the Sensory Benefits of Eye Widening in Fear Expressions. Psychol. Sci. 24, 957–965 (2013). 23620549 10.1177/0956797612464500

[b12] Jack R. E., Garrod O. G. B. & Schyns P. G. Dynamic Facial Expressions of Emotion Transmit an Evolving Hierarchy of Signals over Time. Curr. Biol. 24, 187–192 (2014). 24388852 10.1016/j.cub.2013.11.064

[b13] Smith M. L., Cottrell G. W., Gosselin F. & Schyns P. G. Transmitting and Decoding Facial Expressions. Psychol. Sci. 16, 184–189 (2005). 15733197 10.1111/j.0956-7976.2005.00801.x

[b14] Schyns P. G., Petro L. S. & Smith M. L. Transmission of Facial Expressions of Emotion Co-Evolved with Their Efficient Decoding in the Brain: Behavioral and Brain Evidence. Plos One 4, e5625 (2009). 19462006 10.1371/journal.pone.0005625 PMC2680487

[b15] Benedikt L., Cosker D., Rosin P. L. & Marshall D. Assessing the uniqueness and permanence of facial actions for use in biometric applications. IEEE Trans. Syst. Man. Cybern. A. Syst. Hum. 40, 449–460 (2010).

[b16] Rosenblum L. D., Johnson J. A. & Saldaña H. M. Point-light facial displays enhance comprehension of speech in noise. J. Speech. Hear. Res. 39, 1159–1170 (1996). 8959601 10.1044/jshr.3906.1159

[b17] Kaulard K., Cunningham D. W., Bülthoff H. H. & Wallraven C. The MPI Facial Expression Database — A Validated Database of Emotional and Conversational Facial Expressions. PLoS One 7, e32321 (2012). 22438875 10.1371/journal.pone.0032321 PMC3305299

[b18] Cunningham D. W. & Wallraven C. Dynamic information for the recognition of conversational expressions. J. Vis. 9, 7–7 (2009). 20055540 10.1167/9.13.7

[b19] Ambadar Z., Schooler J. W. & Cohn J. F. Deciphering the enigmatic face the importance of facial dynamics in interpreting subtle facial expressions. Psychol. Sci. 16, 403–410 (2005). 15869701 10.1111/j.0956-7976.2005.01548.x

[b20] Hill H. & Johnston A. Categorizing sex and identity from the biological motion of faces. Curr. Biol. 11, 880–885 (2001). 11516651 10.1016/s0960-9822(01)00243-3

[b21] Yankouskaya A., Humphreys G. W. & Rotshtein P. The processing of facial identity and expression is interactive, but dependent on task and experience. Front. Hum. Neurosci. 8, 1–12 (2014). 25452722 10.3389/fnhum.2014.00920 PMC4231971

[b22] Roark D. A., Barrett S. E., Spence M., Habdi H. & O’Toole A. J. Memory for moving faces: Psychological and Neural Perspectives on the Role of Motion in Face Recognition. Beh. Cognit. Neurosci. Rev. 2, 15–46 (2003). 10.1177/1534582303002001002 17715597

[b23] Xiao N. G. . On the facilitative effects of face motion on face recognition and its development. Front. Psychol. 5, 1–16 (2014). 25009517 10.3389/fpsyg.2014.00633 PMC4067594

[b24] Dobs K. . Quantifying human sensitivity to spatio-temporal information in dynamic faces. Vision Res. 100, 78–87 (2014). 24784699 10.1016/j.visres.2014.04.009

[b25] Ambadar Z., Cohn J. F. & Reed L. I. All Smiles are Not Created Equal: Morphology and Timing of Smiles Perceived as Amused, Polite, and Embarrassed/Nervous. J. Nonverbal Behav. 33, 17–34 (2008). 19554208 10.1007/s10919-008-0059-5 PMC2701206

[b26] Lander K., Christie F. & Bruce V. The role of movement in the recognition of famous faces. Mem. Cog. 27, 974–985 (1999). 10.3758/bf03201228 10586574

[b27] Lander K., Chuang L. & Wickham L. Recognizing face identity from natural and morphed smiles. Q. J. Exp. Psychol. B. 59, 801–808 (2006). 10.1080/17470210600576136 16608747

[b28] Rosenblum L. D. . Visual speech information for face recognition. Percept. Psychophys. 64, 220–229 (2002). 12013377 10.3758/bf03195788

[b29] Luettin J., Thacker N. A. & Beet S. W. Speaker identification by lipreading. Proc. Int. Conf. Spoken Language Processing 1, 62–65 (1996).

[b30] Lander K., Hill H., Kamachi M. & Vatikiotis-Bateson E. It’s not what you say but the way you say it: Matching faces and voices. J. Exp. Psychol.-Hum. Percept. Perform. 33, 905–914 (2007). 17683236 10.1037/0096-1523.33.4.905

[b31] Kamachi M., Hill H., Lander K. & Vatikiotis-Bateson E. ‘Putting the face to the voice’: Matching identity across modality. Curr. Biol. 13, 1709–1714 (2003). 14521837 10.1016/j.cub.2003.09.005

[b32] Back E., Jordan T. R. & Thomas S. M. The recognition of mental states from dynamic and static facial expressions. Vis. Cogn. 17, 1271–1286 (2009).

[b33] Bernstein M. & Yovel G. Two neural pathways of face processing: A critical evaluation of current models. Neurosci. Biobehav. R. 55, 536–546 (2015). 10.1016/j.neubiorev.2015.06.010 26067903

[b34] Cunningham D. W., Kleiner M. & Wallraven C. Manipulating video sequences to determine the components of conversational facial expressions. ACM Trans. Appl. Percept. 2, 251–269 (2005).

[b35] Gur R. C., Sara R., Hagendoorn M. & Marom O. A method for obtaining 3-dimensional facial expressions and its standardization for use in neurocognitive studies. J. Neurosci. (2002). 10.1016/s0165-0270(02)00006-7 11992665

[b36] Curio C. . Semantic 3d motion retargeting for facial animation. ACM Trans. Appl. Percept. 77–84 (2006).

[b37] Kleiner M. Visual stimulus timing precision in psychtoolbox-3: tests, pitfalls and solutions. Perception 39, 189 (2010).

[b38] Luce R. D. A choice theory analysis of similarity judgments. Psychometrika 26, 151–163 (1961).

[b39] Bartlett M. S., Littlewort G. C., Frank M. G. & Lee K. Automatic Decoding of Facial Movements Reveals Deceptive Pain Expressions. Curr. Biol. 24, 738–743 (2014). 24656830 10.1016/j.cub.2014.02.009 PMC4034269

[b40] Macmillan N. A. & Creelman C. D. Detection Theory (Psychology Press, 2004).

[b41] Shannon C. E. A mathematical theory of communication. Bell Syst. Tech. J. 27, 379–423 & 623–656 (1948).

[b42] Quian Quiroga R. & Panzeri S. Extracting information from neuronal populations: information theory and decoding approaches. Nat. Rev. Neurosci. 10, 173–185 (2009). 19229240 10.1038/nrn2578

